# School Botany; or an Explanation of the Characters and Differences of the Principal Natural Classes and Orders of Plants Belonging to the Flora of Europe

**Published:** 1839-10

**Authors:** 


Art. IX.—*School Botany; or an Explanation of the Characters and Differences of the principal Natural Classes and Orders of Plants belonging to the Flora of Europe, in the Botanical Classification of De Candolle.* By John Lindley, ph.d. f.r.s., Professor of Botany in University College.—*London*, 1839. 8vo, pp. 218.

This little work is expressly composed "for the use of students preparing for their matriculation examination in the University of London," but it is admirably adapted for botanical students of all kinds. It is, in fact, a compendious introduction to botany, on the natural system, and is such as might have been expected from the author's great knowledge and long experience as a teacher. We recommend it to every beginner in the study of the natural system of botany, whether young or old, as the best guide in his early progress. The style is simple and clear; and every difficulty in the descriptions is lessened by numerous woodcuts.

